# The putative transporter MtUMAMIT14 participates in nodule formation in *Medicago truncatula*

**DOI:** 10.1038/s41598-023-28160-8

**Published:** 2023-01-16

**Authors:** Kevin Garcia, Kaylee Cloghessy, Danielle R. Cooney, Brett Shelley, Sanhita Chakraborty, Arjun Kafle, Aymeric Busidan, Unnati Sonawala, Ray Collier, Dhileepkumar Jayaraman, Jean-Michel Ané, Guillaume Pilot

**Affiliations:** 1 Department of Crop and Soil Sciences, North Carolina State University, Raleigh, NC 27695-7619 USA; 2 Department of Bacteriology, University of Wisconsin-Madison, Madison, WI 53706 USA; 3 School of Plant and Environmental Sciences, Virginia Tech, Blacksburg, VA 24060 USA; 4 Department of Agronomy, University of Wisconsin-Madison, Madison, WI 53706 USA; 5 Present Address: Department of Biological Sciences, The University of Notre Dame, Notre Dame, IN 46556 USA; 6 Present Address: Molecular Technologies Department, Wisconsin Crop Innovation Center, University of Wisconsin-Madison, Madison, WI 53562 USA

**Keywords:** Plant symbiosis, Rhizobial symbiosis

## Abstract

Transport systems are crucial in many plant processes, including plant–microbe interactions. Nodule formation and function in legumes involve the expression and regulation of multiple transport proteins, and many are still uncharacterized, particularly for nitrogen transport. Amino acids originating from the nitrogen-fixing process are an essential form of nitrogen for legumes. This work evaluates the role of MtN21 (henceforth MtUMAMIT14), a putative transport system from the MtN21/EamA-like/UMAMIT family, in nodule formation and nitrogen fixation in *Medicago truncatula*. To dissect this transporter’s role, we assessed the expression of *MtUMAMIT14* using GUS staining, localized the corresponding protein in *M. truncatula* root and tobacco leaf cells, and investigated two independent *MtUMAMIT14* mutant lines. Our results indicate that MtUMAMIT14 is localized in endosomal structures and is expressed in both the infection zone and interzone of nodules. Comparison of mutant and wild-type *M. truncatula* indicates MtUMAMIT14, the expression of which is dependent on the presence of *NIN, DNF1,* and *DNF2*, plays a role in nodule formation and nitrogen-fixation. While the function of the transporter is still unclear, our results connect root nodule nitrogen fixation in legumes with the UMAMIT family.

## Introduction

Most legume plants have unique evolutionary adaptations to support efficient nitrogen acquisition. Their roots develop a symbiotic relationship with dinitrogen-fixing bacteria called rhizobia forming specialized structures referred to as nodules^1, 2^. Free-living rhizobia produce lipo-chitooligosaccharides, also called Nod factors, that trigger a cascade of molecular responses in the host root^3^. Plant nodules can be classified in two ways, determinate and indeterminate. The meristem of the determinate nodule fully differentiates in other cell types upon maturity. In contrast, the meristem of the indeterminate nodule continues to grow throughout the lifetime of the nodule^4, 5^. *Medicago truncatula* nodules are indeterminate and are composed of five developmental zones^2, 6^, each zone corresponding to the progression of the nodule. The meristematic zone (I) is where new cells that will differentiate into other nodule zones are produced. The infection zone (II) is the location where the rhizobia are released into cells by an endocytosis-like process from the non-walled tips of the infection threads with the simultaneous formation of symbiosomes. The interzone (II-III) allows the bacterial final differentiation into nitrogen-fixing bacteroids. The nitrogen fixation zone (III) is the active part of the nodule where the actual process of fixing nitrogen occurs. The senescence zone (IV) is where the bacteroids degrade and are no longer functional^5, 6^. Finally, in the saprotrophic zone, the degraded cells that harbored symbiosomes earlier are colonized by rhizobia escaping the infection threads^7^.

Amino acids are critical for allocating the organic nitrogen throughout the plant organs and are the primary source of nitrogen provided by the plant to the growing organs^8, 9^. Inorganic sources of nitrogen (nitrate and ammonium) obtained by the plant from the soil are assimilated into organic forms, such as amino acids^10^. From the site of synthesis in the legume root, amino acids enter the xylem for long-distance translocation, via the transpiration stream, to leaves^11^. In the leaves, amino acids might be transiently stored, integrated into vegetative storage proteins, or actively loaded into the phloem for redistribution to sink tissues, like developing root or shoot meristems or seeds^12^. In nodulated amide-centric leguminous plants, atmospheric dinitrogen fixed by bacteroids is converted into ammonia. Ammonia moves to the symbiosome where it is converted into ammonium and transported to the plant compartment, from which amino acids are synthesized^13^. The amino acid cycle in nodulated roots is more complicated than a simple one-way transport from nitrogen-fixing bacteroids to colonized nodules. In addition to their transfer from the bacteroids to the plant cells, plant amino acids may also be needed for the development and function of bacteroids. They are transported via ABC transporters from the plant^14, 15^.

A family of proteins involved in amino acid bi-directional transport identified in plants is the MtN21/EamA-like/UMAMIT (Usually Multiple Acids Move In and Out Transporters) family, which belongs to the drug/metabolite protein transport super family^16, 17^. Only some members have been investigated in *Arabidopsis thaliana* so far, including WAT1/AtUMAMIT05, AtSiAR1/AtUMAMIT18, AtUMAMIT14 and AtUMAMIT24/25, and all exhibit amino acid export or bidirectional activity^17–22^. Although the first member of the UMAMIT family, Nodulin 21 (MtN21), was initially identified in *M. truncatula* as a gene induced by nodulation^23^, no other members have been described in a nitrogen-fixing plant thus far. We conducted a phylogenetic analysis to establish the extent of the UMAMIT family within *M. truncatula*. We determined the temporal and cellular expression patterns exhibited by *MtN21* (henceforth *MtUMAMIT14*) in *M*. *truncatula* roots colonized or not by nitrogen-fixing bacteria, characterized with promoter-GUS approach the activity and tissue specificity of the *MtUMAMIT14* promoter, and determined the subcellular localization of MtUMAMIT14. To dissect the role of MtUMAMIT14, both in nodule formation on *M*. *truncatula* roots and nodule nitrogen fixation performance, we investigated two independent *MtUMAMIT14* mutant lines compared to wild-type plants.

## Results

### Phylogenetic analysis of UMAMIT proteins from Medicago truncatula

**Figure 1:**
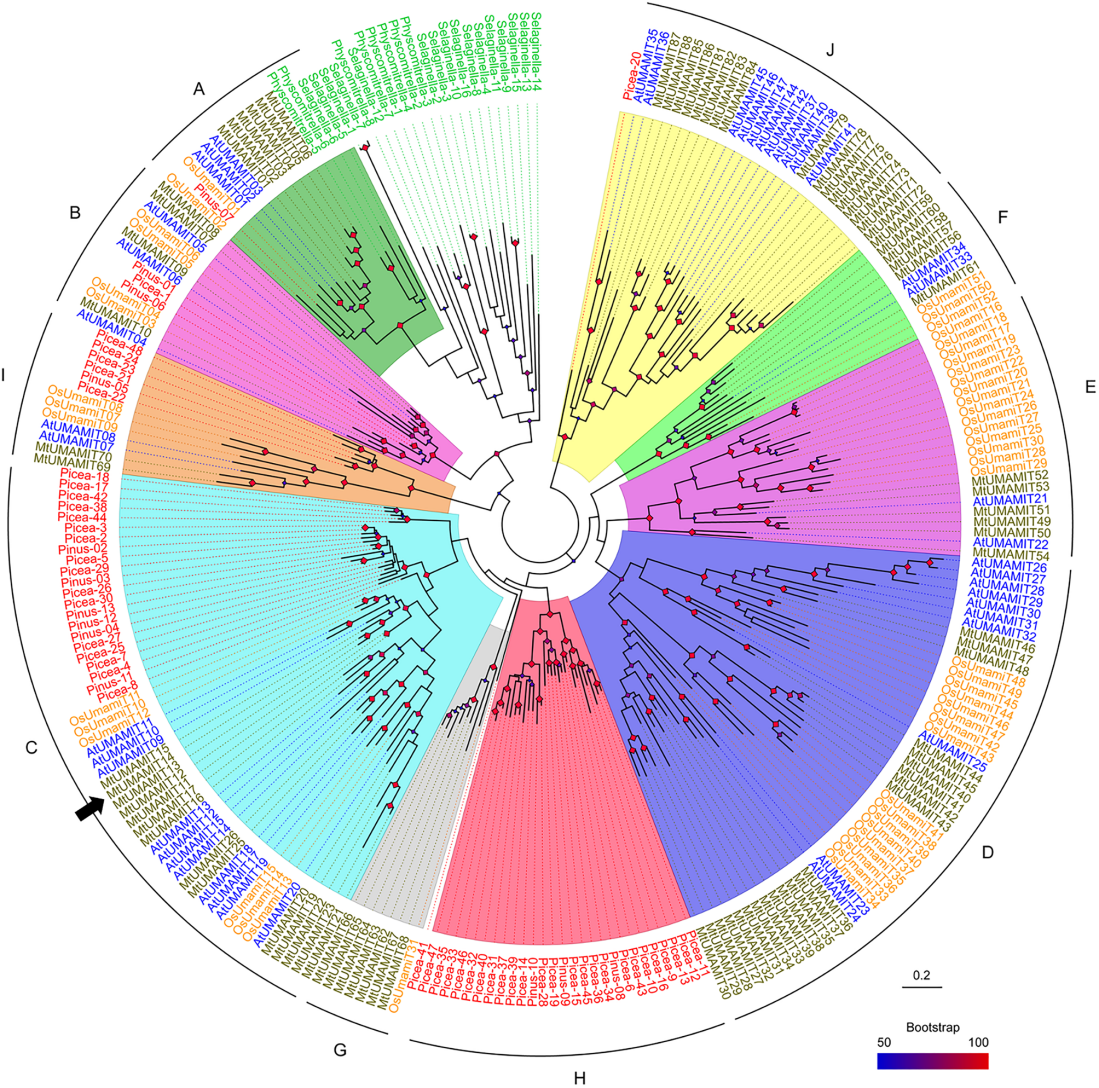
Phylogenetic tree of UMAMIT proteins from seven selected species. The sequences from 46 UMAMITs from *A. thaliana* (AtUMAMITs), 88 UMAMITs from *M. truncatula* (MtUMAMITs), 53 from *Oryza sativa* (OsUMAMITs), 16 from *Selaginella moellendorffii* (Selaginella), 8 from *Physcomitrella patens* (Physcomitrella), 13 from *Pinus pinaster* (Pinus), and 48 from *Picea abies* (Picea) were used for phylogenic reconstruction, using the Maximum Likelihood method. The tree with the highest log-likelihood is shown. Bootstrap values are indicated by blue (50%) to red (100%) diamonds on each node; nodes with less than 50% support are not shown. A black arrow indicates the position of MtUMAMIT14. The clades (A to J) correspond to the clades identified using a phylogenetic reconstruction from 1466 UMAMIT sequences belonging to 38 species. Scale: number of substitutions per site.

Using the sequences of an *A. thaliana* gene from each of the UMAMIT clades as a template, we identified and collected 88 UMAMIT loci from *M. truncatula* (Table S1). Phylogenetic reconstruction (Fig. 1) showed that MtUMAMIT14 (Mt4.0: Medtr3g012420 / Mt5.0: MtrunA17_Chr3g0081511) belongs to Clade C and is phylogenetically close to AtUMAMIT09 (At5G07050) from *A. thaliana*. The clades (A to J) correspond to the clades identified using a phylogenetic reconstruction from 1466 UMAMIT sequences belonging to 38 species^17^.

### MtUMAMIT14 is expressed in the infection zone and interzone of M. truncatula nodules

**Figure 2:**
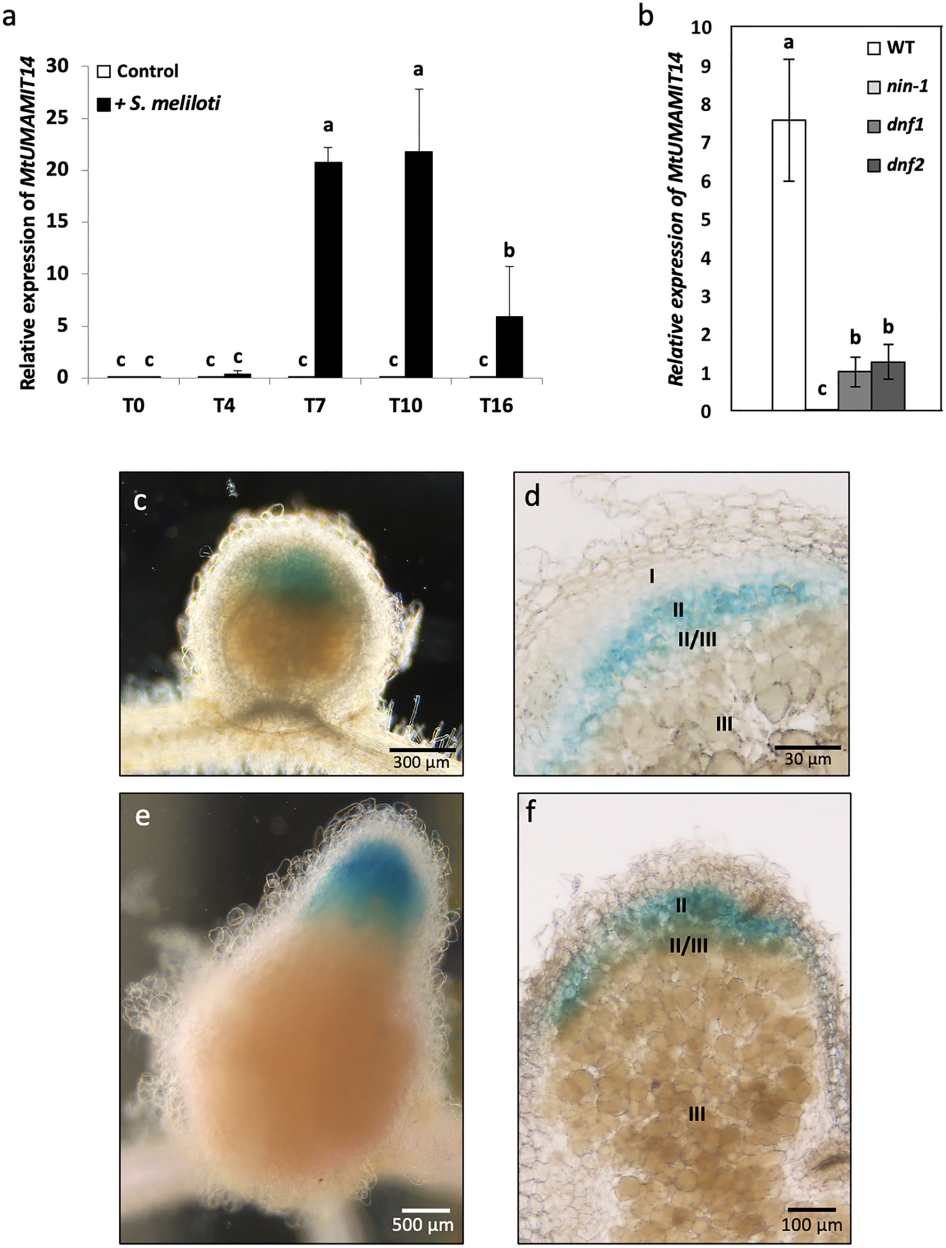
Expression pattern of *MtUMAMIT14* in *Medicago truncatula*. (**a**) The relative expression of *MtUMAMIT14* was determined by RT-qPCR in plants inoculated with *S. meliloti* after 0, 4, 7, 10, or 16 dpi (grey bars) or kept non-inoculated (white bars) relative to the expression of *MtTef1α*. (**b**) The expression of *MtUMAMIT14* was determined in *dnf1*, *dnf2*, *nin-1* mutants, and wild-type (WT) of *M. truncatula* 10 dpi with *S. meliloti*. Different letters indicate significant differences between treatments according to one-way (**b**) or two-way (**a**) ANOVA followed by LSD post hoc tests (*P* < 0.05). n = 4. (**c**–**f**). *PromMtUMAMIT14::GUS* fusion revealed that *MtUMAMIT14* was expressed in zone II and inter-zone II/III of *M. truncatula* nodules. Nodules were stained 10 (**c**,**d**) and 16 (**e**,**f**) dpi.

*M. truncatula* nodules emerge from the roots approximately 7 days after inoculation with *Sinorhizobium meliloti*, but nitrogen fixation starts around 10 days after inoculation. Seedlings were inoculated with *S.* *meliloti* or kept non-inoculated to evaluate the expression pattern of *MtUMAMIT14*. Roots were collected at 0, 4-, 7-, 10-, and 16-days post-inoculation (dpi), and the relative expression level of *MtUMAMIT14* was determined by RT-qPCR (Fig. 2a). Control plants exhibited minimal to no expression of *MtUMAMIT14* throughout the evaluation range of 16 dpi. However, plants inoculated with *S. meliloti* exhibited *MtUMAMIT14* expression beginning at 4 dpi, though not statistically different from control plants. At 7 and 10 dpi, inoculated plants showed a significant up-regulation of *MtUMAMIT14* relative to the control*.* Expression of *MtUMAMIT14* was reduced at 16 dpi compared to the 7 and 10 dpi time points, while remaining significantly higher compared to non-inoculated plants (Fig. 2a). These results match what can be found in the “*M. truncatula* RNA-seq Gene Expression Atlas Project” dataset^24^, in which *MtUMAMIT14* expression was strong in nodulated roots, and rather limited in mycorrhizal roots, compared to other organs (Fig. S1).

The transcription factor NIN is required in various stages of nodule development^25, 26^. To determine if the symbiotic induction of *MtUMAMIT14* expression requires *NIN,* we quantified its expression in the inoculated roots of wild-type *vs.* the *NIN* loss-of-function (*nin-1*) mutant plants that cannot initiate nodules. *MtUMAMIT14* expression was not detected in the *nin-1* background, suggesting that *NIN* might directly or indirectly participate for symbiotic *MtUMAMIT14* expression (Fig. 2b).

After emerging from the host root, *M. truncatula* nodules keep differentiating further. *Defective in Nitrogen Fixation* (*DNF*) genes encode crucial proteins for rhizobial differentiation before nitrogen fixation^27^. The loss-of-function mutants show defects in rhizobial differentiation, although the earlier stages of development are relatively unaffected. The expression of *MtUMAMIT14* was significantly reduced in the *dnf1* and *dnf2* mutants at 10 dpi, suggesting that proper *MtUMAMIT14* expression in the nodules is contingent upon successful bacteroid differentiation (Fig. 2b).

To determine the spatial expression pattern of *MtUMAMIT14*, composite plants, which have wild-type shoots and transgenic roots^28–30^, were generated using hairy root-inducing *Agrobacterium rhizogenes*, which contains a binary vector carrying the *PromMtUMAMIT14::GUS* fusion. Once transformed roots developed, we inoculated them with *S. meliloti.* Five plants were taken for the GUS assays at 10 and 16 dpi, longitudinal cross-sections of nodules were examined, and representative images were displayed in Fig. 2. At both time points in whole nodules, GUS staining was detected only at the nodule tip (Fig. 2c,e). GUS staining was observed in the infection zone (zone II) and the interzone II/III at both 10 and 16 dpi (Fig. 2d,f).

### MtUMAMIT14 protein is localized at endosomal structures of Medicago and tobacco cells

We investigated the subcellular localization of MtUMAMIT14 *in planta* by expressing a YFP-tagged version of MtUMAMIT14 in *Nicotiana benthamiana* epidermal cells. Three days after injection, samples were observed by confocal microscopy, and fluorescence was detected in punctate structures that typically correspond to endosomes (Fig. S2).

**Figure 3:**
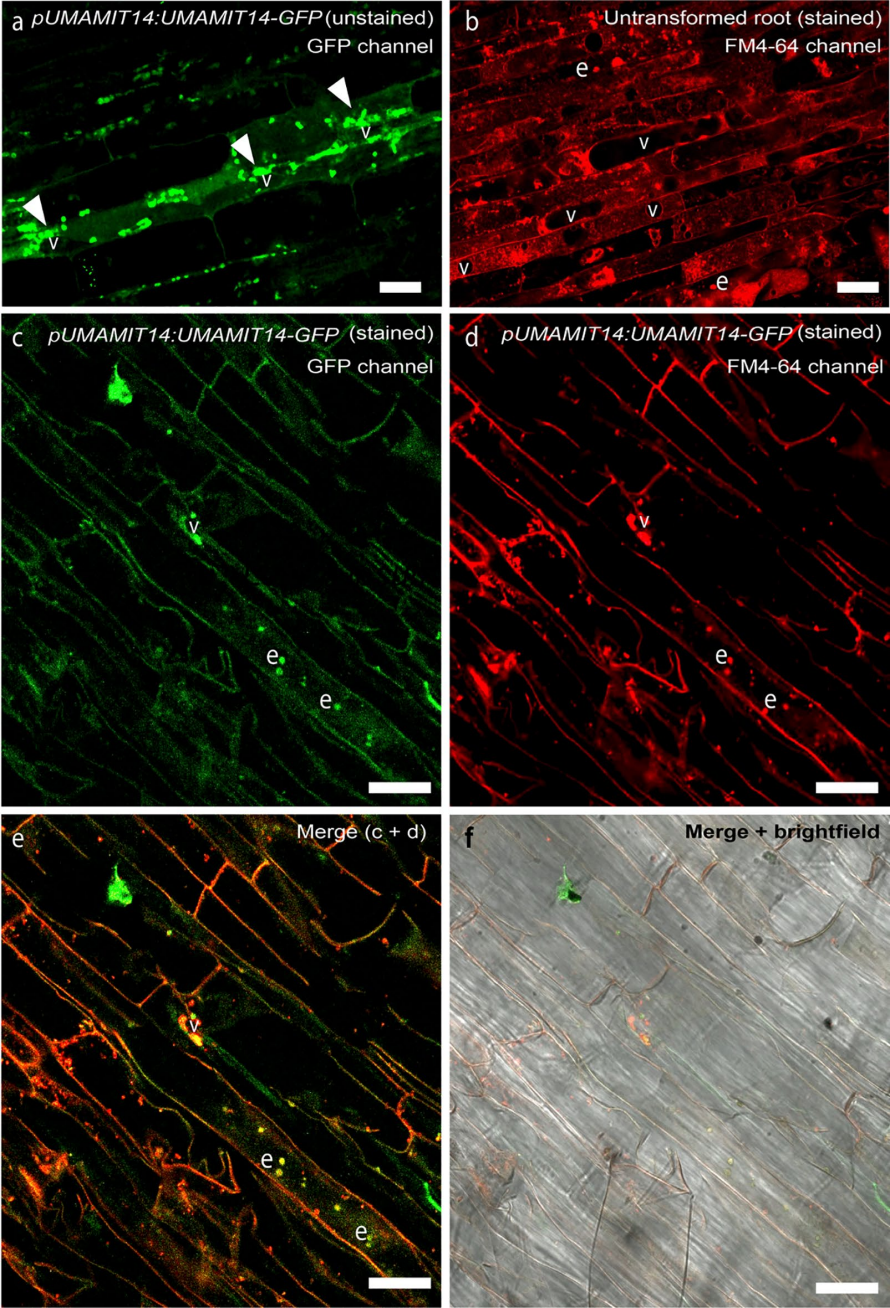
Subcellular localization of *MtUMAMIT14* in *Medicago truncatula* roots. (**a**) *pMtUMAMIT14:MtUMAMIT14-GFP* localization in unstained roots. (**b**) FM4-64-stained, untransformed roots. (**c**–**f**) Roots transformed with *pMtUMAMIT14:MtUMAMIT14-GFP* after staining with FM4-64 in various channels; (**c**) GFP, (**d**) FM4-64, (**e**) overlay, and (**f**) overlay in brightfield. The arrowheads in (**a**) indicate punctate GFP localization. “e” denotes endosome. “v” denotes vacuole. Scale bar = 20 µm. n = 6.

To confirm this observation*,* we also expressed a C-terminal GFP-tagged version of MtUMAMIT14 under its native promoter (*pMtUMAMIT14:MtUMAMIT14-GFP*) in *Medicago truncatula* composite plants*.* The transformed roots displayed GFP fluorescence puncta of various sizes in the interior of the cells, likely representing individual and clustered endosomes^31^, and often appearing around the vacuoles (Fig. 3a). To better resolve the localization, we stained the roots with FM4-64, which stains various membranes of *M. truncatula* cells depending upon the duration of staining^31^. After three hours of incubation, the FM4-64 signal was observed from the plasma membrane, the tonoplast, and various components of the cellular trafficking network (Fig. 3b). When *pMtUMAMIT14:MtUMAMIT14-GFP*-expressing roots were stained, GFP/FM4-64 co-localization was prominently observed in endosomes (Fig. 3c–f). Since the pMtUMAMIT14:MtUMAMIT14-GFP punctate structures were often observed associated with the tonoplast and never with the plasma membrane, MtUMAMIT14 could be targeted to the vacuoles via the endosomes.

### MtUMAMIT14 mutants are affected in nodule formation and nitrogen fixation

**Figure 4:**
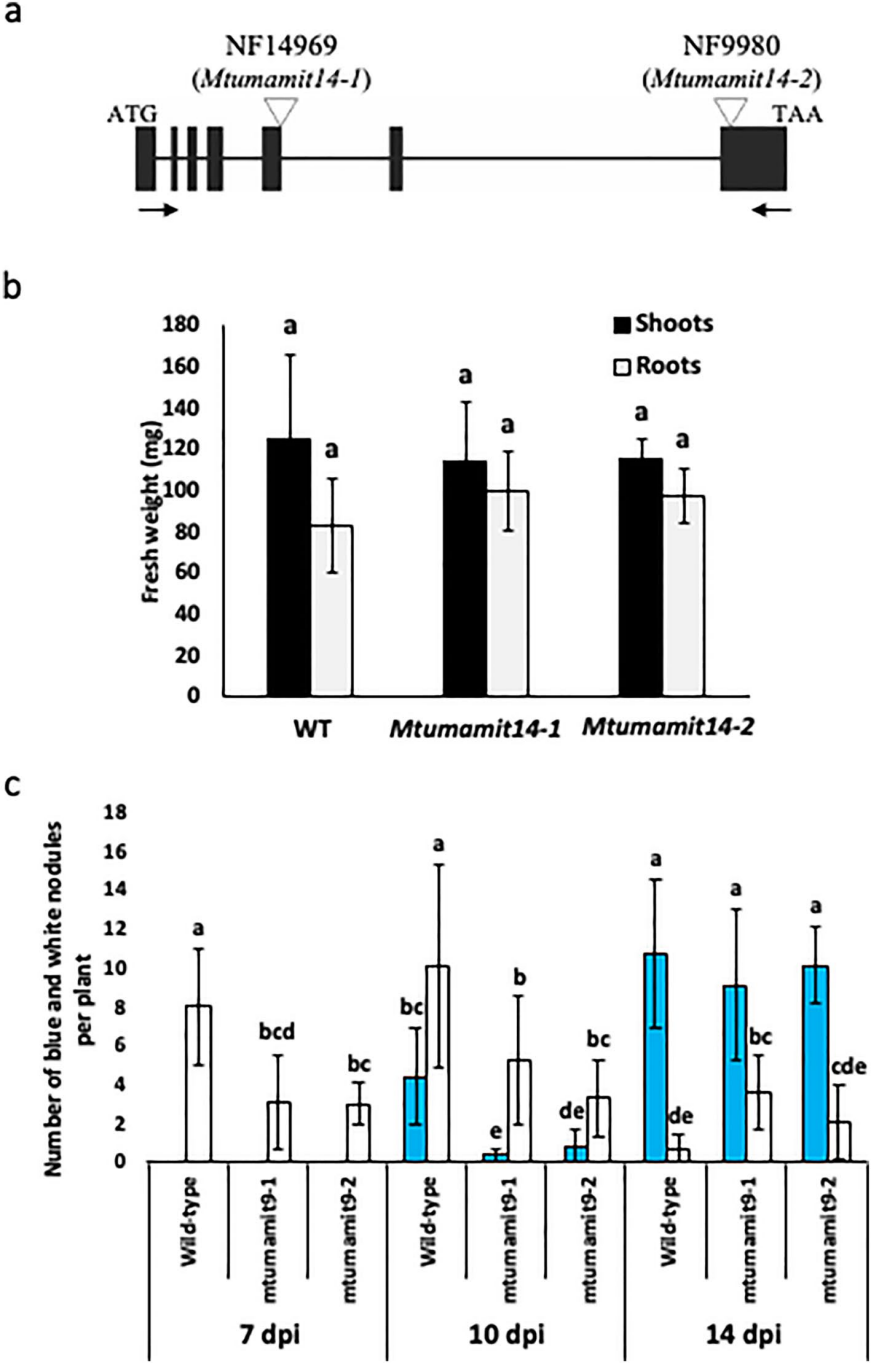
Mutation in *Medicago truncatula* MtUMAMIT14 transporter results in fewer nodules and nitrogen fixation. (**a**) The structure of the *MtUMAMIT14* gene shows the retrotransposon *Tnt1* insertions of the *Mtumamit14-1* and *Mtumamit14-2* mutant alleles. The black arrows indicate the primers used for RT-qPCR validation presented in Fig. S3. (**b**) The fresh weight of shoots and roots of nodulated Wild-type and *Mtumamit14-1/2 M. truncatula* was determined 10 dpi. (**c**) The number of nodules per plant was determined in Wild-type and *Mtumamit14-1/2 M. truncatula* 7, 10, and 14 dpi with the strain of *S. meliloti* containing the *nifH::GUS* fusion. Different letters indicate significant differences between treatments according to two-way ANOVA followed by LSD post hoc tests (*P* < 0.05). n = 6–9.

According to the sequences available in public databases, the composition of this gene is seven exons forming the *MtUMAMIT14* cDNA sequence (Fig. 4a). In NF14969 and NF9980, *Tnt1* was inserted in the fifth and seventh exon, respectively. NF14969 and NF9980 lines were named *Mtumamit14-1*, and *Mtumamit14-2*, respectively (Fig S3). To validate these lines, RT-PCRs were performed using primers amplifying the entire coding sequence, and showed that *Mtumamit14-1* expressed a truncated version of *MtUMAMIT14*, and no transcript was detected in *Mtumamit14-2* (Fig. S4). Sequencing of amplifications observed in Fig. S4 in wild-type and *Mtumamit14-1* lines revealed that around 150 bp were missing in the *MtUMAMIT14* sequence of *Mtumamit14-1*, certainly resulting in an impaired or missing protein (Fig. S5). The MtUMAMT14 mutants were phenotypically characterized by analyzing plant biomass, nodule numbers, and nitrogen fixation. Aboveground and belowground fresh weights were determined on wild-type and both *Mtumamit14-1* and *Mtumamit14-2* mutant lines 10 days after rhizobial inoculation because this time point corresponds to when *MtUMAMIT14* expression was the highest (Fig. 2a). No significant differences were noted for fresh biomass between wild-type and mutant lines (Fig. 4b).

Since *M. truncatula* nodules are discernible on the root at about 7 dpi and nitrogen fixation apparent by 10 dpi, we evaluated the ability of *Mtumamit14-1* and *Mtumamit14-2* mutants to develop not only nodules but functional nodules using a bacterial symbiont expressing the *nifH::GUS* construct. Since bacterial *nifH* gene is involved in nitrogenase production, following its expression using a β-glucuronidase (*GUS*) reporter gene allowed the observation of fixing (“blue”) and non-fixing (“white”) nodules for each plant. Thus, the number of fixing and non-fixing nodules was recorded at 7, 10, and 14 dpi in wild-type and mutant lines (Fig. 4c). At 7 dpi, there was a significant reduction of about 50% in the total number of nodules produced in *Mtumamit14-1* and *Mtumamit14-2* lines compared to wild-type seedlings. At this time point, GUS staining results were negative for nodules developing on roots of wild-type and *Mtumamit14* lines, indicating the presence of non-fixing nodules only. At 10 dpi, the number of nodules was increased in all genotypes but remained lower in the two mutant lines as compared to wild-type (Fig. 4c). Both numbers of fixing and non-fixing nodules were significantly reduced in *Mtumamit14-1* and *Mtumamit14-2* mutants compared to the wild-type plants. At 14 days, a similar number of fixing nodules was recorded in all lines. However, the numbers of non-fixing nodules were significantly higher in both mutants as compared to wild-type (Fig. 4c).

**Figure 5:**
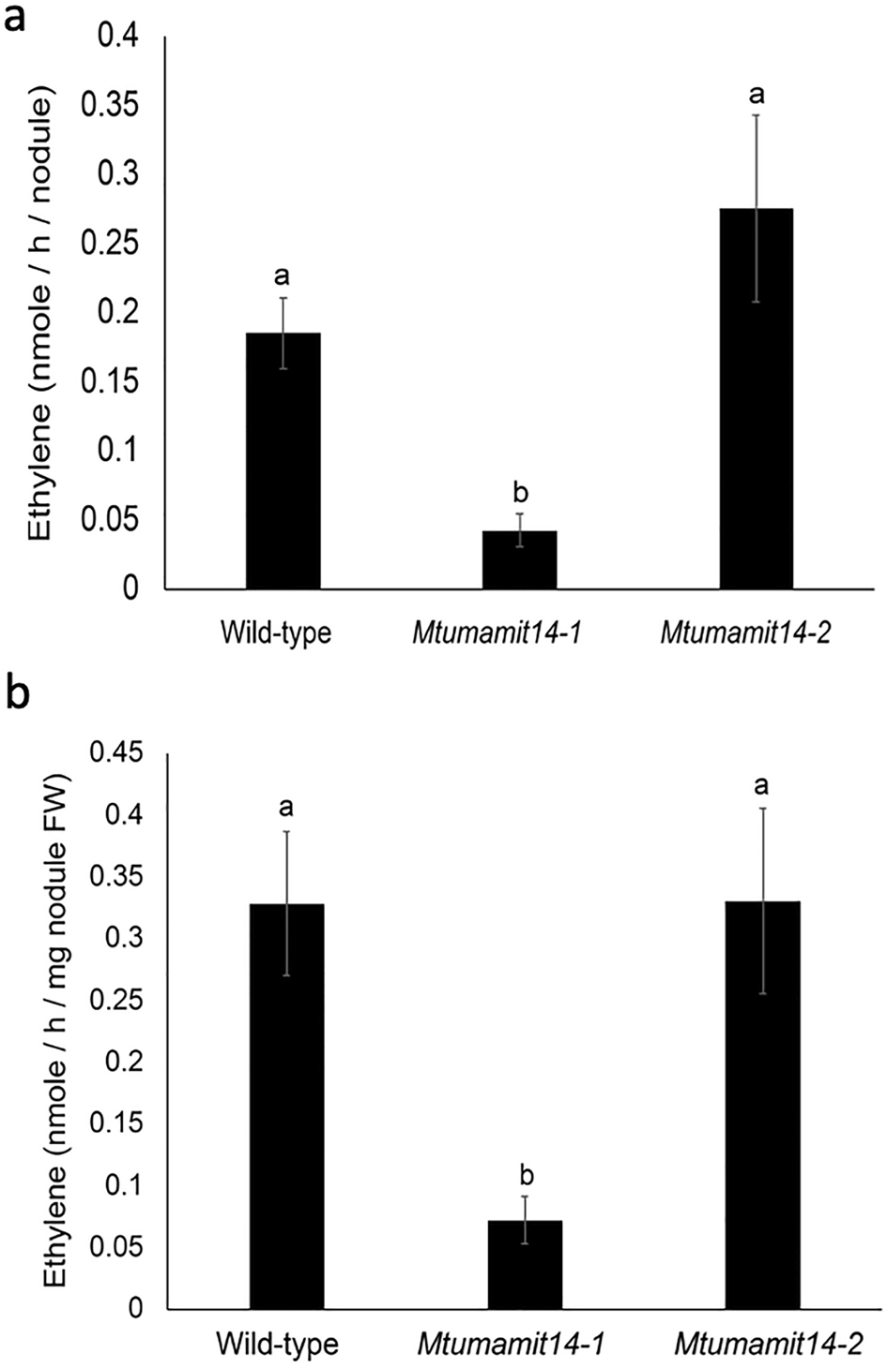
Mutation in *Medicago truncatula* MtUMAMIT14 transporter results in reduced nitrogen fixation. Ethylene (C_2_H_4_) production was recorded 19 h after injection of 10% acetylene on 14-days-old wild type (WT) and *Mtumamit14-1/2* seedlings. (**a**) Ethylene production per hour measured by ARA and normalized to the number of nodules per plant. (**b**) Ethylene production per hour normalized to nodule fresh weight (mg) per plant. Different letters indicate significant differences between treatments according to one-way ANOVA followed by LSD post hoc tests (*P* < 0.05). n = 11–15.

To assess the impact of MtUMAMIT14 on nitrogen fixation, the ability of both *Mtumamit14-1* and *Mtumamit14-2* to fix nitrogen was determined. Acetylene reduction assays were conducted on entire plants at 14 dpi in wild-type and mutant lines, and the amount of ethylene produced was recorded 19 h later (Fig. 5). Several of the nodules on *Mtumamit14-2* roots were visibly more pinkish and elongated compared to the wild-type nodules. Nitrogenase activity was significantly lower in *Mtumamit14-1* compared to the wild-type and *Mtumamit14-2* backgrounds, normalized both to nodule number and weight (Fig. 5a,b). The differences in phenotypes in *Mtumamit14-1* and *Mtumamit14-2* lines could be attributed to the relative positions and impacts of retrotransposon insertion (Fig. 4a, S5).

Altogether, our results indicate that MtUMAMIT14 inactivation has a dramatic impact on the nodule formation kinetic and may eventually decrease nitrogen fixation in *M. truncatula* plants.

### The functional properties of MtUMAMIT14 could not be revealed in yeast

So far, all active UMAMITs have displayed amino acid export transport properties^18, 21, 22^. To test whether MtUMAMIT14 can export amino acids, the amino acid secretion assay we previously developed in *Saccharomyces cerevisiae* was used^18^. MtUMAMIT14 was expressed in the 22Δ10α strain (MATα gap1-1 put4-1 uga4-1 can1::HisG lyp1-alp1::HisG hip1::HisG dip5::HisG gnp1Δ agp1Δ ura3-1^18^) that is not able to grow on any proteinogenic amino acid except for arginine as the sole nitrogen source^18^, and the medium collected after about one day was analyzed for amino acid content. Contrary to AtUMAMIT14, 18, 23, 24, and 25^18, 19^, no accumulation of amino acids at levels higher than the empty vector could be detected (Fig. S6). Additionally, 22Δ10α was transformed with plasmid derivatives of pDR196, leading to (1) the co-expression of amino acid permease AtAAP3 and AtUMAMIT14 carrying knockout mutations (AtUMAMIT14-GGVV^17^), AtUMAMIT14, MtUMAMIT14, or the sucrose transporter AtSUC2, or (2) the expression of MtUMAMIT14, MtUMAMIT14-GFP, AtUMAMIT14 or AtAAP3 (Fig. S7a). The yeast expressing AtAAP3 alone were able to grow on minimum medium containing 3 mM Gly as the sole nitrogen source. Co-expression of AtAAP3 and mutant AtUMAMIT14 or AtSUC2 did not affect growth, proving that co-expression of membrane protein devoid of amino acid export activity did not affect overall uptake. On the contrary, co-expression of the amino acid exporter AtUMAMIT14 reduced growth, thus uptake. MtUMAMIT14 was not able to reduce growth of yeast expressing AtAAP3, nor able to sustain growth on itself (similar to AtUMAMIT14). This suggests that MtUMAMIT14 does not transport amino acid in yeast. Similar results were obtained for all other amino acids (Ala, Asp, Asn, Citrulline, GABA, Gln, Glu, Ile, Leu, Met, Ornithine, Phe, Pro, Ser, Thr, Val), supplied at 3 or 12 mM. These negative results could not be explained by a low protein expression in yeast because western blot experiments with transformed yeast revealed the presence the GFP alone or the MtUMAMIT14-GFP construct (Fig. S7b). Therefore, a localization in internal membrane structures might prevent export activity at the plasma membrane.

## Discussion

A major path for acquisition of atmospheric nitrogen by certain leguminous plants involves nodule formation in which nitrogen fixation occurs within a symbiotic partner bacterium. Recently, several studies have explored the role played by plant transport proteins in legume nodulation and nitrogen fixation^32–39^. This work aims to investigate another putative transport protein, MtUMAMIT14, in nodule development and nitrogen fixation in the model legume *M. truncatula*.

Some members of the UMAMIT family were previously investigated in the non-fixing model plant *A. thaliana*^17^. For example, AtUMAMIT18/SiAR1 transports amino acids into the phloem towards sinks and connects xylem and phloem transfer in roots^21, 40^, and AtUMAMIT11, 14, 24, 25, 28, and 29 play essential roles in seed, embryo development, and export in roots^18, 22^. *CsUMAMIT9* from the tea plant exhibited differential expression under conditions of high aluminum accumulation^41^. Our search of the *M. truncatula* genome identified 88 genes encoding proteins with high sequence similarity to UMAMITs. MtUMAMITs were found in the 9 clades containing angiosperm species, with approximately twice as many genes in each of the clades as *A. thaliana*, consistent with the whole genome duplication which occurred ~ 58 Myr ago in the ancestor of *M. truncatula*^42^. Interestingly, seven MtUMAMITs and one OsUMAMIT belong to Clade G, a clade in which no *A. thaliana* sequence was found. This clade may contain transporters that are important for the physiological functions of *M. truncatula* and rice not found in *A. thaliana*, e.g., mycorrhizal symbiosis^43^. Additionally, previous RNA sequencing data on *M. truncatula* revealed that, although lowly expressed, *MtUMAMIT14* transcripts were also detected in roots colonized by an arbuscular mycorrhizal fungus (Fig. S1). However, additional work will be needed to test this hypothesis, and further experiments are still required to decode the role of the other UMAMIT members in *M. truncatula* and determine if phylogenetic similarities between both model plants can predict similar functions.

Subcellular localization approach is crucial to predict the function of transport proteins. For example, protoplasts were used to localize AtUMAMIT24 in the tonoplast of seeds and AtUMAMIT25 at the plasma membrane^19^. Other UMAMIT transporters from *A. thaliana* were localized at the plasma membrane^21, 22^, tonoplast^20, 44^, and ER membrane^45^. As no UMAMIT member has been investigated in *M. truncatula* so far, we decided to focus on MtUMAMIT14 since it was initially identified as being induced by nodulation^23^, a clue for possible involvement in nodulation nitrogen-fixing symbiosis. Subcellular localization in both tobacco leaves and *M. truncatula* roots revealed MtUMAMIT14 presence in endosomal structures, suggesting a potential role in trafficking for the formation of infection threads and/or bacteroids as described for MtSYP132 and MtVAMP721^46, 47^.

To unravel the role of MtUMAMIT14 in nodule formation and nitrogen fixation, we assessed the expression of *MtUMAMIT14* in wild-type and mutant plants affected in nodulation, and we investigated two *MtUMAMIT14 Tnt1* insertion lines. Nodulated wild-type roots exhibited an increased expression of *MtUMAMIT14*, particularly at 7 and 10 dpi. The location of *MtUMAMIT14* expression in the nodule corresponded to zone II and the interzone II-III. This expression pattern and the targeting of MtUMAMIT14 proteins to the endosome suggest an active role of this transporter in rhizobial infection and/or differentiation of symbiosomes, but not in the later stages of symbiosis and during nitrogen fixation. Although this expression in zones II and II-III is partly in line with what was observed previously, it was also found that *MtUMAMIT14* can be expressed in the nitrogen fixing zone of *M. truncatula* nodules (Fig. S1, zone III)^48^. This discrepancy can be due to different environmental conditions, or difference in nodule maturity. The transcription factor NODULE INCEPTION (NIN) plays a central role in nodule organogenesis and regulation in *M. truncatula*^49–52^. *NIN* is expressed in the root cortex and rhizodermis and impacts the number of functional nodules^25, 26, 53^. Evaluating *NIN* role in the functionality of nodules showed that this particular gene can influence the formation of nodules along with the infection potential of the nitrogen-fixing bacteria^51^. Characterization of the mutant *nin-1,* unable to form nodules, revealed its pivotal role in developing infection threads and initiating nodule primordia^50^. *MtUMAMIT14* expression is undetectable in the *nin-1* background, confirming that the presence of nodules is required for MtUMAMIT14 function in *M. truncatula*. Furthermore, the expression of *MtUMAMIT14* was investigated in two *dnf* mutants (*defective in nitrogen fixation*) that form non-fixing nodules^54^. The *dnf* mutants fail to support nitrogen fixation, limiting the developmental process at different stages. As such, they can be classified into different groups. Among them, *dnf1* and *dnf2* are ranked in the same group that allows for bacterial infection within the nodule’s inner cortex^54^. In both *dnf* backgrounds, *MtUMAMIT14* was still expressed but significantly lower than wild-type plants (Fig. 3b). This confirms that MtUMAMIT14 functions upstream from nitrogen fixation and might be involved in infection and/or differentiation events. Understanding what substrate MtUMAMIT14 can transport is crucial to appreciate its role in pre-fixing steps. For example, the ABC transporter AatA transports aspartate and is necessary for the bacteroid to fix nitrogen in pea and alfalfa efficiently^14, 55^. Another study evaluated branched-chain amino acid transport (LIV) as an essential mechanism in peas but indicated that this process was not present in *M. truncatula*^15^. Our attempts to functionally characterize MtUMAMIT14 in yeast deficient in amino acid transport were inconclusive, likely due to the localization of the corresponding proteins in endosomal structures. However, further experiments in other heterologous systems are needed to determine which amino acids MtUMAMIT14 can transport and if amino acids could be provided to the bacteria for their differentiation into bacteroids, or from bacteroids to plant cells.

The phenotyping of two independent *Mtumamit14* mutants revealed a delayed nodule formation of *nifH::GUS* expressing nodules at 7 and 10 dpi. The nodule formation delay associated in both mutants to affected bacterial colonization and cellular differentiation, suggesting a role of MtUMAMIT14 in these nodule development processes. However, at 14 dpi, mutants displayed also increases of the total nodule numbers per plant affecting the number of nodules not expressing *nifH::GUS*. This suggested that new waves of nodule formation were activated in the mutants in response to plant N deficit due to fix- nodulation^56–58^. Indeed, measurement of nitrogen fixation activity in the *Mtumamit14.1* mutant revealed a lower nitrogen fixation activity as compared to the wild-type consistent with the hypothesis of a nitrogen acquisition default in the mutant resulting in a stimulation of nodule formation. However, a lower nitrogen fixation activity was not observed at 14 dpi in the nodules of the second mutant *Mtumamit14.2*, which was not in agreement with the hypothesis. Nevertheless, it could be speculated that a transient default of nitrogen fixation due to the nodule formation delay of this mutant might be sufficient to stimulate new waves of nodule formation, but this remains to be experimentally demonstrated. Intriguingly, a similar increase in the nodule number was observed on transgenic hairy roots of soybean in which RNAi was used to knock-down the expression of the ureide transporters GmUPS1-1 and GmUPS1-2^36^. Further investigations are still needed to clarify the role of these transporters in these nodulation stimulation phenotypes.

## Methods

### Biological material and growth conditions

Seeds of *M. truncatula* ecotype R108 wild type were used in this study, and *Mtumamit14-1* and *Mtumamit14-2* mutant plants were formally identified by the Noble foundation. *nin-1*, *dnf1*, and *dnf2* mutants were described previously^51, 54^. This work does not involve the collection of plant or seed specimens and complies with relevant institutional, national, and international guidelines and legislation.

Seeds were acid-scarified with concentrated H_2_SO_4_ for 7 min, surface-sterilized with commercial bleach (8% w/v of Cl) for 2 min, rinsed in sterile water, and kept in water for 1 h. Sterilized seeds were vernalized at 4 °C for 5 days on 1% agar supplemented with 1 µg.ml^-1^ of gibberellic acid and allowed to germinate overnight at room temperature. The germinated seedlings were placed for two weeks onto modified Fahräeus medium before their transfer to pots, as described previously^59, 60^. Seedlings were transferred to pots filled with Turface (EP Minerals 7941 Minerals Safety Absorbent), watered with tap water, inoculated 2 days later with rhizobia, and harvested at 0, 4, 7, 10, and 14 or 16 dpi depending on the experiment. *S. meliloti* 1021 or the CSB357 strain containing a *nifH::GUS* fusion^61^ were used to inoculate the seedlings. *A. rhizogenes* strain MSU440 harboring the transcriptional fusion vector was used to transform *M. truncatula* roots^29^. *Agrobacterium tumefaciens* strain AGL-1 was used for agroinfiltration of *Nicotiana benthamiana* leaves. Acetylene reduction measurements were carried out on 10-day old plants with nodules as described previously^62^.

A screening of the *M. truncatula Tnt1* mutant collection at the Noble Foundation identified *MtUMAMIT* mutant lines NF14969 and NF9980^63, 64^. Flanking sequence tags (FSTs) corresponding to the *Tnt1* insertion sites were available for these two lines (http://bioinfo4.noble.org/mutant/index.php). The mutant lines were validated by RT-PCR after root RNA extraction of plants 10 days after rhizobial inoculation using the corresponding forward and reverse primers (5′-ATGGGCGCTGAAAAGCC-3′ and 5′-TGTTTGATTCTTCTTTCTGATCCAAC-3′, respectively).

### Cloning

To study the spatial expression pattern of *MtUMAMIT14*, a binary vector containing a transcriptional unit with the *PromMtUMAMIT14::GUS* fusion was assembled using modules from both the MoClo Toolkit and the MoClo Plant Parts Kit^65^ (www.addgene.org). Position 1 of the binary vector (proximal to the T-DNA Left Border) was assigned to a fluorescent marker to aid in the non-destructive identification of transgenic roots of *M*. *truncatula*. A Golden Gate Level 1 reaction was conducted to assemble in pICH47732 (plasmid Level 1, position 1 forward; pL1F-1) a transcriptional unit featuring the 2 × enhanced Cauliflower Mosaic Virus 35S promoter (in combination with the Tobacco Mosaic Virus Ω translational enhancer) (pICH51288) driving expression of tandem Tomato^66, 67^ (ST1290-5) with polyadenylation under control of the *A.* *tumefaciens Nopaline synthase* (*NOS*) terminator (pICH41421). The *MtUMAMIT14* promoter *GUS* transcriptional unit was assigned to position 2 of the T-DNA, proximal to the T-DNA Right Border. The *MtUMAMIT14* promoter was shielded from the impact of the bidirectional CaMV35S enhancers because both transcriptional units were oriented in the same direction, with the 5′ end of the *MtUMAMIT14* promoter abutted to the 3′ end of the NOS terminator of the fluorescent marker cassette. The *MtUMAMIT14* promoter (1963 bp) was domesticated to remove BsaI, BbsI, and Esp3I restriction sites, synthesized and cloned by Synbio Technologies (http://www.synbio-tech.com/) into pICH41295 (the Level 0 Promoter + 5′ UTR (pL0-P5U) acceptor) backbone to obtain a Golden Gate compatible level 0 P5U module. A Golden Gate Level 1 reaction was conducted with pICH47742 (pL1F-2) with the aforementioned Level 0 P5U *MtUMAMIT14* promoter, as well as pICH75111 and pICH41432 level 0 modules containing the *uidA* coding DNA sequence (CDS1) and *A*. *tumefaciens Octopine synthase* (*OCS*) terminator modules, respectively. Each Golden Gate Level 1 assembly was performed in a PCR tube with a 15 µL mixture containing 100 ng of each level 0 module, 100 ng of the corresponding level 1 backbone, 1.5 µl of 10X NEB T4 buffer, 0.15 µl of bovine serum albumin (BSA), 1 µl (10 units) of BsaI, 1 µl (400 units) of T4 DNA ligase, and Milli-Q water. Reactions were incubated in a thermocycler oscillating between 37 °C (optimum temperature for the Type IIS restriction endonuclease) for 3 min and 16 °C (optimum temperature for T4 DNA ligase) for 4 min. These temperature conditions were repeated for 25 cycles. The reaction was terminated by incubation at 50 °C for 5 min (to inactivate the T4 DNA ligase), and subsequently, 80 °C for 5 min (to inactivate the Type IIS restriction endonuclease). 2 µl of the finished Golden Gate reaction products were used to transform chemically competent cells of *Escherichia coli* strain DH5α. Transformed colonies were selected on solid LB medium supplemented with 100 μg/ml of ampicillin, 100 μg/ml of IPTG, and 40 μg/ml of X-gal. The finished binary vector was constructed in a Golden Gate Level 2 reaction in which 100 ng of the MoClo Level 2 acceptor pAGM4673 (RK2 replicon, similar to pBIN19) was mixed with the aforementioned Level 1 transcriptional units containing the 2 × 35S::TdTomato::Ter-Nos (100 ng) and Prom-MtUMAMIT14::GUS::Ter-OCS (100 ng) constructs were mixed with, 100 ng of the end-linker module pICH41744 (endlinker position 2 (pELE-2); mediates the connection of pL1F-2 to pAGM4673), 1.5 µl of 10X NEB T4 buffer, 0.15 µl of BSA, 1 µl of BbsI, 1 µl of T4 DNA ligase (2000 units), and Milli-Q water, then subjected to the same Golden Gate temperature cycling protocol as was used for Level 1 assembly. To construct the plasmid to express the MtUMAMIT14-YFP chimeric protein constitutively, the CDS of *MtUMAMIT14* was cloned into the pDONRZeo Gateway entry vector by BP reaction, sequenced, and mobilized into pC4H-WY^68^, leading to the expression of the fusion protein specifically in leaf epidermis cells. To express MtUMAMIT14 in yeast, the *MtUMAMIT14* CDS cloned in pDONRZeo was mobilized into the vector pDR196^69^ in which the gateway cassette had been inserted downstream from the PMA1 promoter.

### β-glucuronidase (GUS) activity

Nodules from *M. truncatula* transgenic roots expressing the *Prom-MtUMAMIT14::GUS::Ter-OCS* construct and those expressing the *nifH::GUS* fusion were collected at 7, 10, and 14 days post-inoculation. Plants resulted from five independent transformation events since only roots were transformed independently for each plant using *A. rhizogenes*, and all plants displayed similar specific GUS staining. Around 50 µm of longitudinal sections were obtained using a Vibratome and stained as described previously^70^. Observations of the cross-sections used a Leica DMi1 microscope and a Leica MC120 HD camera using the Leica provided software, and representative images are shown.

### Sub-cellular localization in Medicago truncatula roots

Standard Golden Gate modular cloning was performed to generate *pMtUMAMIT14:MtUMAMIT14-GFP*^65^*.* Domesticated parts, where required, were synthesized from Synbio Technologies. The following level-0 parts were combined in the dig-lig reaction: *MtUMAMIT14* promoter (1.9 kb upstream of the transcription start site), *MtUMAMIT14* CDS without the stop codon, the C-terminal *mEGFP* (Addgene pJOG176), CaMV35S 3′ UTR -terminator (Addgene pICH41414), and the level-1 acceptor (Addgene pICH47811). The *pMtUMAMIT14:MtUMAMIT14-GFP* cassette so generated, along with the level-1 cassette constitutively expressing tdTOMATO-ER (*AtUBI10* (*At4g05320.2*) promoter-5' UTR + tdTOMATO-ER + 3′ UTR-NOS terminator (Addgene pICH41421) were combined with the level-2 linker (Addgene pICH41744) and the level-2 empty backbone (Addgene pAGM4673). The resulting binary vector was introduced into *A. rhizogenes* MSU440 via electroporation.

*M. truncatula* radicles were transformed with *A. rhizogenes* as previously described^29^. The roots were screened for tdTOMATO-ER fluorescence three weeks after transformation under a Leica stereomicroscope and stained with FM4-64 as previously described^31^. The roots were incubated in the dark for three hours.

The sub-cellular localization of pMtUMAMIT14:MtUMAMIT14-GFP was studied using a Zeiss 780 Laser Scanning Confocal Microscope. The Argon laser at 488 nm was used to excite GFP and FM4-64. GFP emission was recorded at 500–550 nm. To minimize the overlap with the tdTOMATO-ER emission spectrum, FM4-64 emission was captured between 698–750 nm. The.czi files were processed using Fiji.

### Agroinfiltration in Nicotiana benthamiana leaves

The MtUMAMIT14-YFP construct was introduced and expressed in leaves of 5-week-old *Nicotiana benthamiana* plants as described^71^. Briefly, the *Agrobacterium* cultures were washed twice in 10 mM MgCL_2_, 100 µM acetosyringone, and cells containing the expression cassettes for MtUMAMIT14-YFP and p19^72^ were mixed and diluted to a final OD_600_ of 0.2 and 0.05, respectively. The solution was infiltrated into the spongy parenchyma of leaves, and the lower epidermis was observed three days later by confocal microscopy using a Zeiss LSM 800 microscope. YFP emission was recorded at 530–560 nm.

### Expression analysis of MtUMAMIT14

We determined the transcript levels of *MtUMAMIT14* in *M. truncatula* seedlings inoculated by *S. meliloti* 1021 at 0, 4, 7, 10, and 16 dpi and in *nin-1*, *dnf1*, and *dnf2* mutants at 10 dpi by RT-qPCR. All following steps concerning RNA extractions, cDNA synthesis, and qPCR amplifications were performed as described in^73^ unless stated otherwise. Briefly, root samples (with or without nodules) were homogenized using liquid nitrogen, and total RNAs were extracted using the PureLinkRNA Mini Kit (Thermo Fisher Scientific, Waltham, MA, USA), treated with TURBO DNase (Thermo Fisher Scientific), and quantified by a NanoDrop ND‐1000spectrophotometer (Thermo Fisher Scientific). cDNAs were synthesized from 400 to 600 ng of DNase‐treated RNAs using the RNA Maxima First Strand cDNA Synthesis Kit with dsDNase (Thermo FisherScientific) and diluted with RNase‐free water to a final concentration of 20 ngμl^-1^. RT-qPCRs were performed using the Bio-Rad CFX96 real-time PCR system with the CFX Manager v3.0 software (Bio-Rad). Reactions were performed in 96-well plates using SsoAdvanced Universal SYBR Green Supermix (Bio-Rad), 10 nM of *MtUMAMIT14* forward, and reverse specific primers (5′-ATAGCTCCGTTCGCCATTATC-3′ and 5′-CCCAAGCAAAGCTAGGAGTAA-3′, respectively), and 1:20 (v/v) cDNA:water. The PCR conditions were as follows: 50 °C for 2 min; 95 °C for 15 min; 40 cycles at 95 °C for 10 s, 60 °C for 15 s, and 72 °C for 20 s; dissociation at 95 °C for 15 s; 60 °C for 15 s; and 95 °C for 15 s. We used MtTef1α as a reference gene^74^, and the expression coefficients were calculated using the 2^−ΔΔCt^ method^75^. The results are based on four biological replicates and three technical replicates. The specificity and efficiency of primer pairs were confirmed by analysis of dissociation curves (65 to 95 °C) and serial dilution, respectively.

### Nitrogenase activity

Nitrogenase activity in wild-type, *Mtumamit14-1,* and *Mtumamit14-2* plants with nodules was determined by acetylene reduction assay at 14 dpi. Seedlings were collected, the number of nodules per plant were counted, and the whole seedlings were placed in 10 ml glass vials filled with 1 ml of sterilized water. Vials were injected with 10% acetylene (Airgas) and incubated for 19 h at room temperature. Control vials were prepared without any plant material and with or without acetylene. Ethylene production was quantified by injecting 1 ml of the air phase on a Gas Chromatography (GC-2010 Shimadzu) equipped with an Rt-Alumina BOND/KCL column (Restek). At the end of the experiment the nodule weight was recorded, and the ethylene production per hour was normalized either to the number of nodules per plant, or nodule fresh weight.

### Phylogenetic tree construction

Using the sequence of each of 8 UMAMIT clades (*i.e.* AtUMAMIT01, 04, 07, 09, 15, 21, 23, 28, 33, 40) as a template, a total of 88 UMAMIT proteins from *M. truncatula* were retrieved from the *M.* *truncatula* A17 r5.0 genome (https://medicago.toulouse.inra.fr/). The sequence of the 88 *Medicago truncatula*, 46 *Arabidopsis thaliana*, 53 *Oryza sativa*, 16 *Selaginella moellendorffii*, 8 *Physcomitrella patens*, 13 *Pinus pinaster,* and 48 *Picea abies* (see ^17^ for the sequences other than *Medicago*) were aligned in MEGA 11 software^76^ using the Muscle algorithm with default parameters. All incomplete sequences and pseudogenes were identified and removed, and the remaining sequences (264) realigned as above. The sequences from the C- and N-termini and the variable central loop of the proteins were removed from the alignment, and tree reconstruction was performed using Maximum Likelihood in MEGA using the JTT matrix-based model^77^, and branch support was determined by running 1000 bootstraps. All positions with less than 95% site coverage were eliminated, and there were 294 positions in the final dataset. The resulting tree was edited in FigTree 1.4.4.

### Expression in yeast for functional characterization

*S. cerevisiae* strain 22∆10α (MATα gap1-1 put4-1 uga4-1 can1::HisG lyp1-alp1::HisG hip1::HisG dip5::HisG gnp1Δ agp1Δ ura3-1^18^) was transformed with the MtUMAMIT14 expression construct using the PEG/LiAc method^78^. Yeast cells were grown in 2 ml liquid minimum medium containing 2.5 mM (NH_4_)_2_SO_4_ at 30 °C at a starting OD_600_ of 0.05 for 22 h. OD_600_ of yeast cultures were measured and supernatants collected by running the cultures through a Millipore filter using MultiScreen HTS Vacuum Manifold (Millipore) first, and then through a 10 kDa exclusion membrane (Ultracell 10, Millipore) by centrifugation at 3,000 g for 90 min at room temperature. Amino acids in the medium were detected by UPLC and quantified as described^79^.

Additionally, more than 50 colonies obtained from a transformation event were pooled and grown at 30 °C for 3 days on SD medium. Cells were resuspended in 500 µl sterile water at an OD600 = 1, and a tenfold dilution was made in water. Five µl of each dilution were then spotted on solid, freshly made minimal medium (yeast nitrogen base without ammonium and amino acids, 50 mM K citrate, pH 6.3, 2% glucose, 1.5% Oxoid Agar Bacteriological), supplemented with 3 or 12 mM amino acid, or 2.5 mM (NH_4_)2SO_4_ as the sole nitrogen source. Cells were grown at 30 °C and plates were scanned every 3 days for 9 days. Control strains expression AtUMAMIT14 and AtUMAMIT14-GGVV (knock-out mutation) were previously generated^17^.

### Western blot

*S. cerevisiae* strains transformed with an empty vector, the GFP alone, or the MtUMAMIT14-GFP construct were grown overnight in 5 ml SD medium, harvested by centrifugation resuspended in 500 µl breaking buffer (50 mM sodium phosphate pH7.4, 1 mM EDTA, 5% glycerol, 1 × complete and 1 M DTT), and about 500 µl of acid-washed 1 mm glass beads. The mix was vortexed for 30 s, cooled down on ice for 30 s, and this cycle was repeated 6 times. After addition of 1 µl 10% SDS, the cells were vortexed once more, and spun at 14,000 × g for 20 min at 4 °C. The supernatant was collected, and 15 µl were separated on a Novex 4–16% SDS-MOPS-MES acrylamide gel according to manufacturer’s instructions. After transfer on a nitrocellulose membrane, proteins were detected using an anti-GFP primary antibody (1:5000; Rockland, CAT 600–401-215) and a goat-anti-rabbit secondary antibody (1:5000, BioRad), by chemiluminescence.

### Statistical analyses

Data are presented as the mean of four to nine replicates, depending on the experiment. Differences between averages were analyzed by one-way or two-way ANOVA, depending on the experiment, followed by LSD post hoc tests.

## Supplementary Information

Supplementary Table S1.

Supplementary Figures.
