# Supplementary material for: The putative transporter MtUMAMIT14 participates in nodule formation in Medicago truncatula
Source: Sci Rep. 2023 Jan 16;13:804. doi: 10.1038/s41598-023-28160-8 (PMC9842706; doi:10.1038/s41598-023-28160-8)
Supplement: Supplementary file 2 — Supplementary Figures. [file 41598_2023_28160_MOESM2_ESM.pptx]

## Slide 1
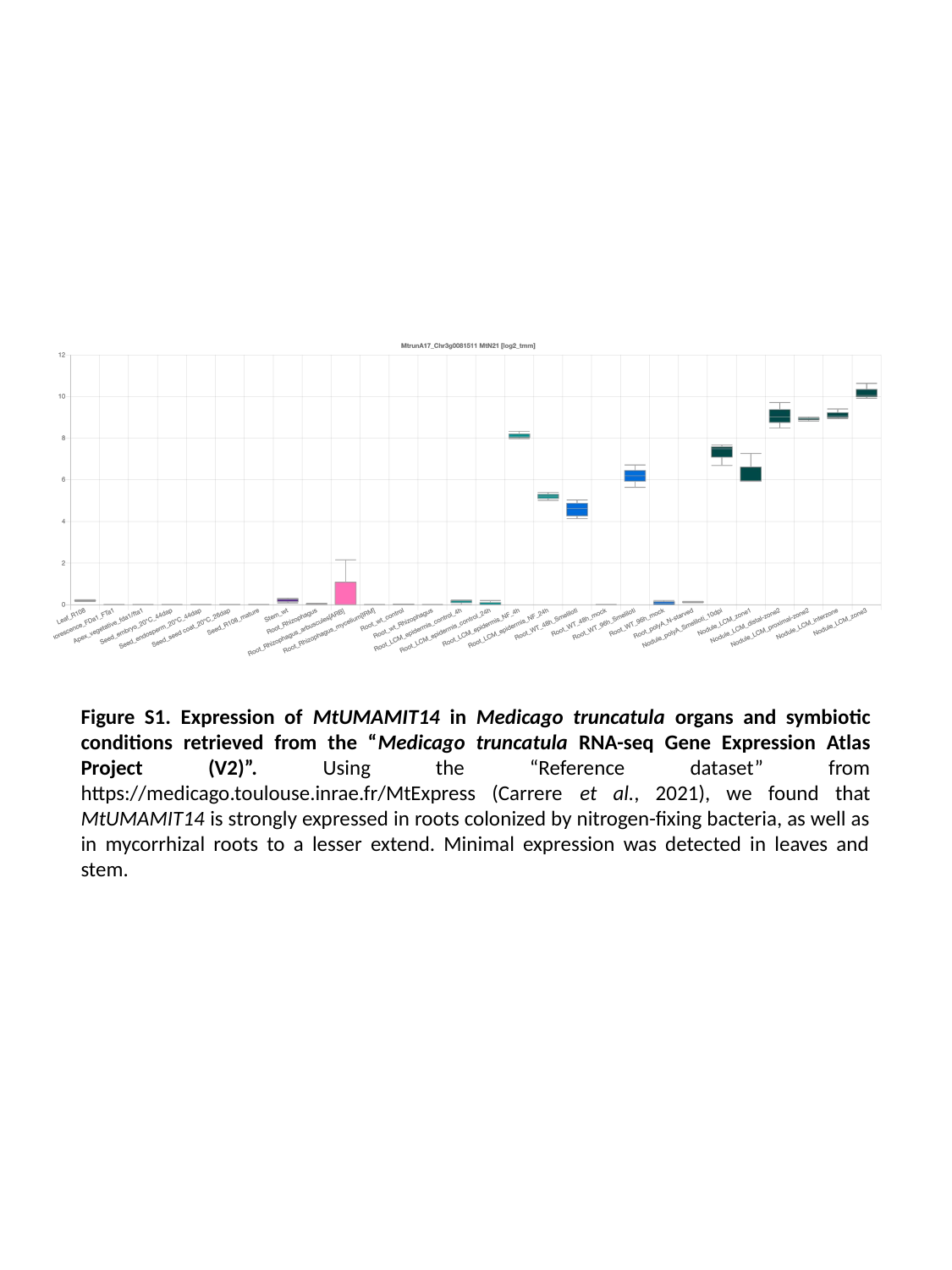

Figure S1. Expression of MtUMAMIT14 in Medicago truncatula organs and symbiotic conditions retrieved from the “Medicago truncatula RNA-seq Gene Expression Atlas Project (V2)”. Using the “Reference dataset” from https://medicago.toulouse.inrae.fr/MtExpress (Carrere et al., 2021), we found that MtUMAMIT14 is strongly expressed in roots colonized by nitrogen-fixing bacteria, as well as in mycorrhizal roots to a lesser extend. Minimal expression was detected in leaves and stem.

## Slide 2
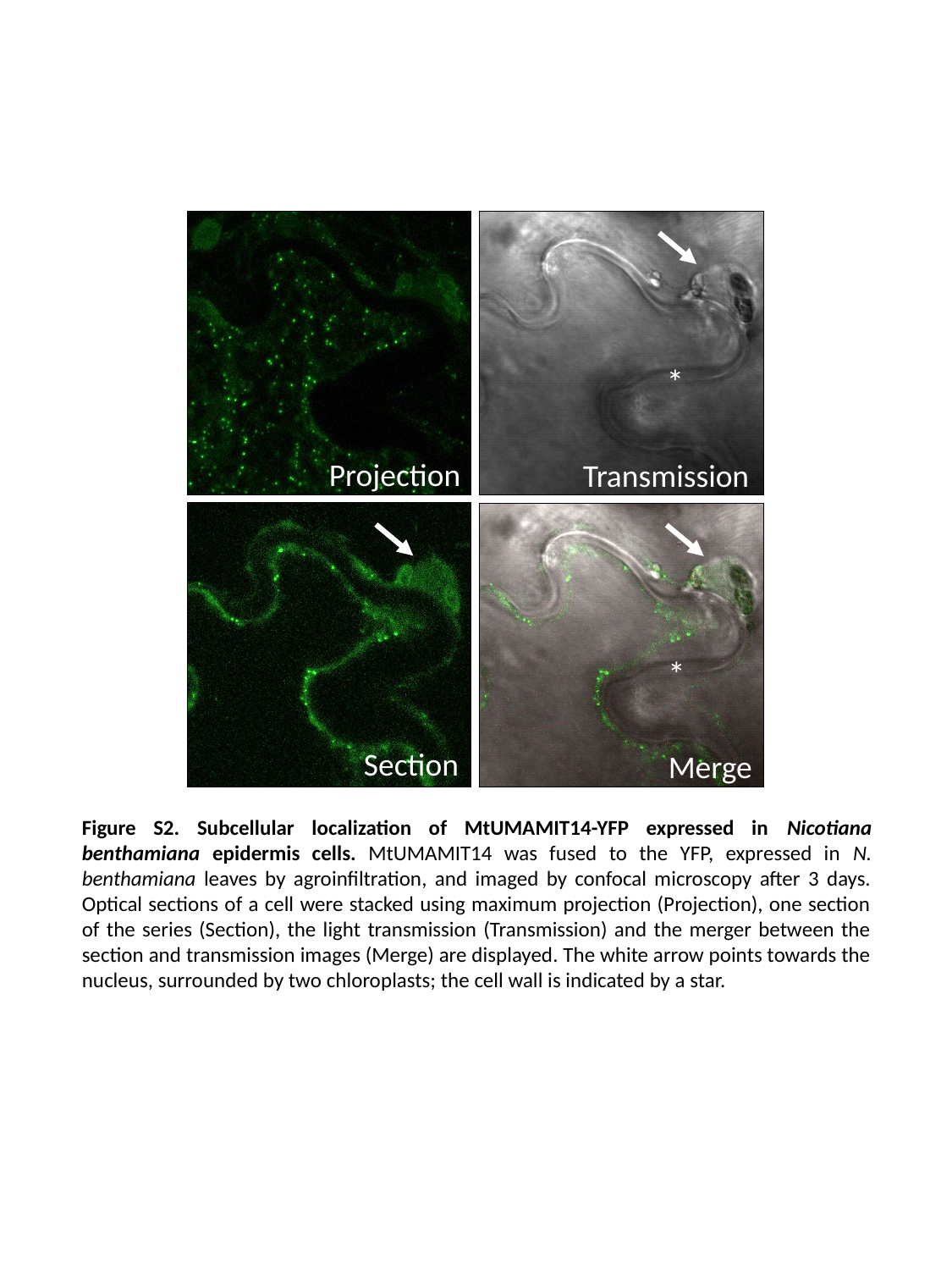

*
Projection
Transmission
*
Merge
Section
Figure S2. Subcellular localization of MtUMAMIT14-YFP expressed in Nicotiana benthamiana epidermis cells. MtUMAMIT14 was fused to the YFP, expressed in N. benthamiana leaves by agroinfiltration, and imaged by confocal microscopy after 3 days. Optical sections of a cell were stacked using maximum projection (Projection), one section of the series (Section), the light transmission (Transmission) and the merger between the section and transmission images (Merge) are displayed. The white arrow points towards the nucleus, surrounded by two chloroplasts; the cell wall is indicated by a star.

## Slide 3
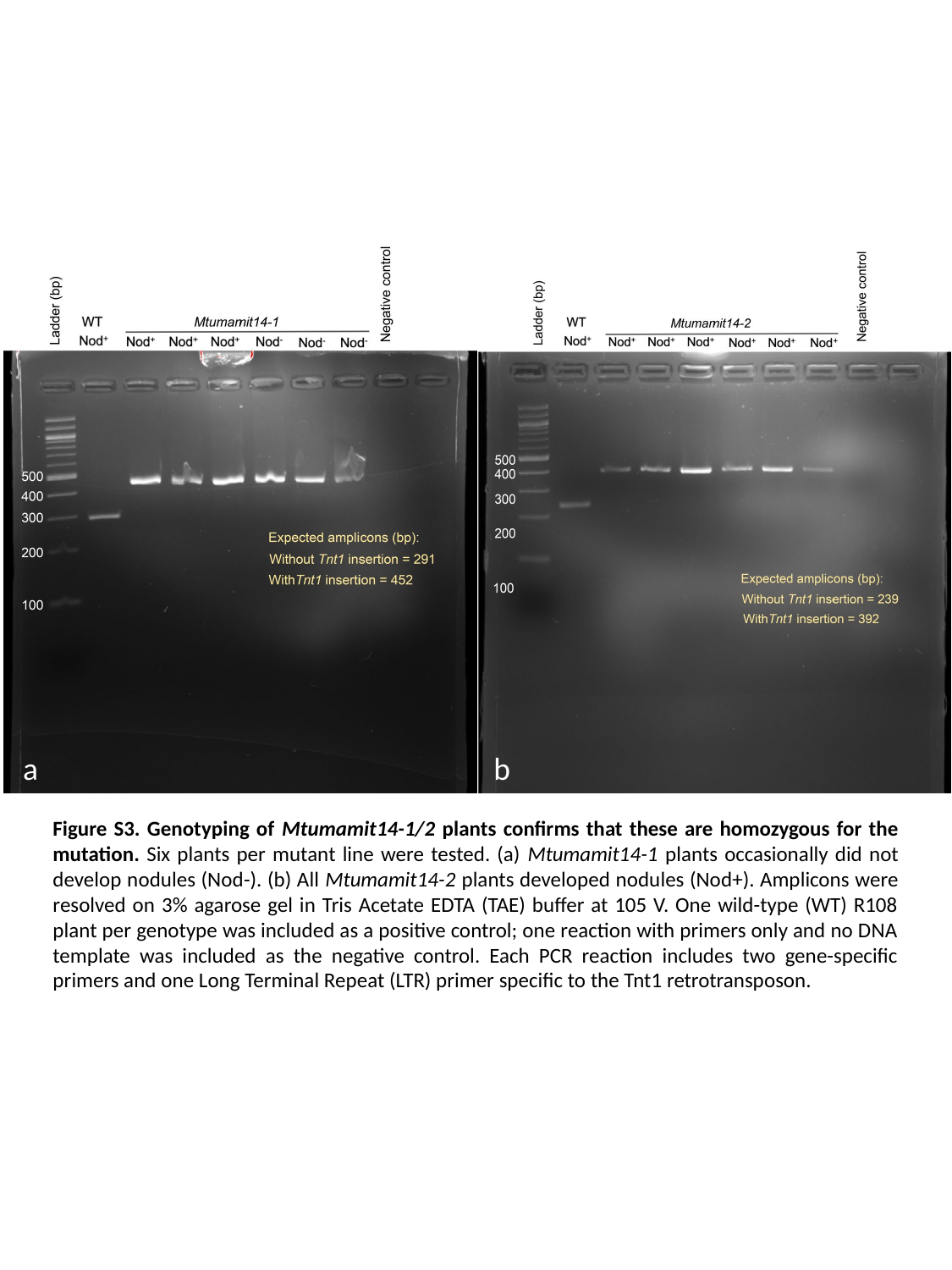

a
b
Figure S3. Genotyping of Mtumamit14-1/2 plants confirms that these are homozygous for the mutation. Six plants per mutant line were tested. (a) Mtumamit14-1 plants occasionally did not develop nodules (Nod-). (b) All Mtumamit14-2 plants developed nodules (Nod+). Amplicons were resolved on 3% agarose gel in Tris Acetate EDTA (TAE) buffer at 105 V. One wild-type (WT) R108 plant per genotype was included as a positive control; one reaction with primers only and no DNA template was included as the negative control. Each PCR reaction includes two gene-specific primers and one Long Terminal Repeat (LTR) primer specific to the Tnt1 retrotransposon.

## Slide 4
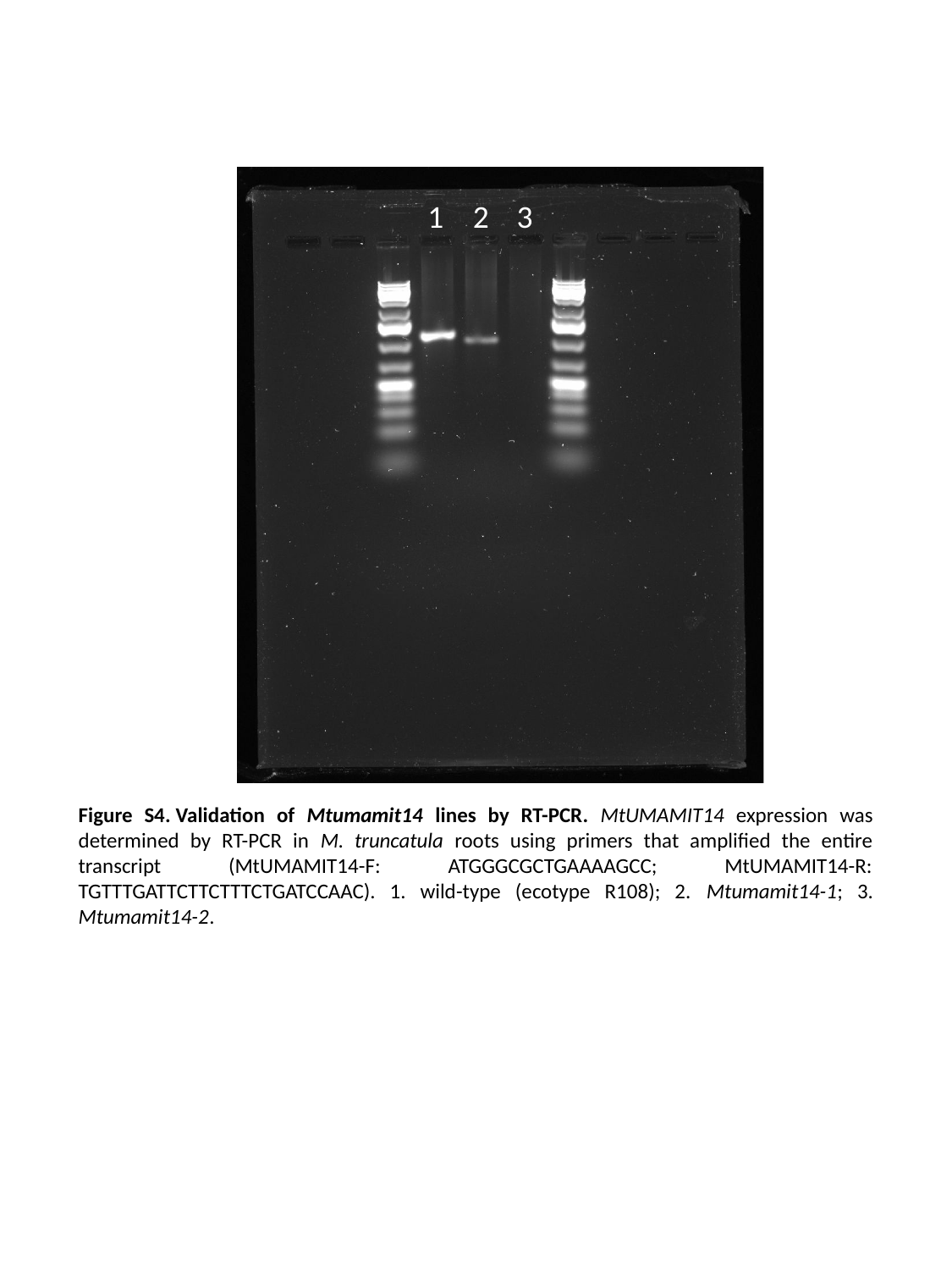

1
2
3
Figure S4. Validation of Mtumamit14 lines by RT-PCR. MtUMAMIT14 expression was determined by RT-PCR in M. truncatula roots using primers that amplified the entire transcript (MtUMAMIT14-F: ATGGGCGCTGAAAAGCC; MtUMAMIT14-R: TGTTTGATTCTTCTTTCTGATCCAAC). 1. wild-type (ecotype R108); 2. Mtumamit14-1; 3. Mtumamit14-2.

## Slide 5
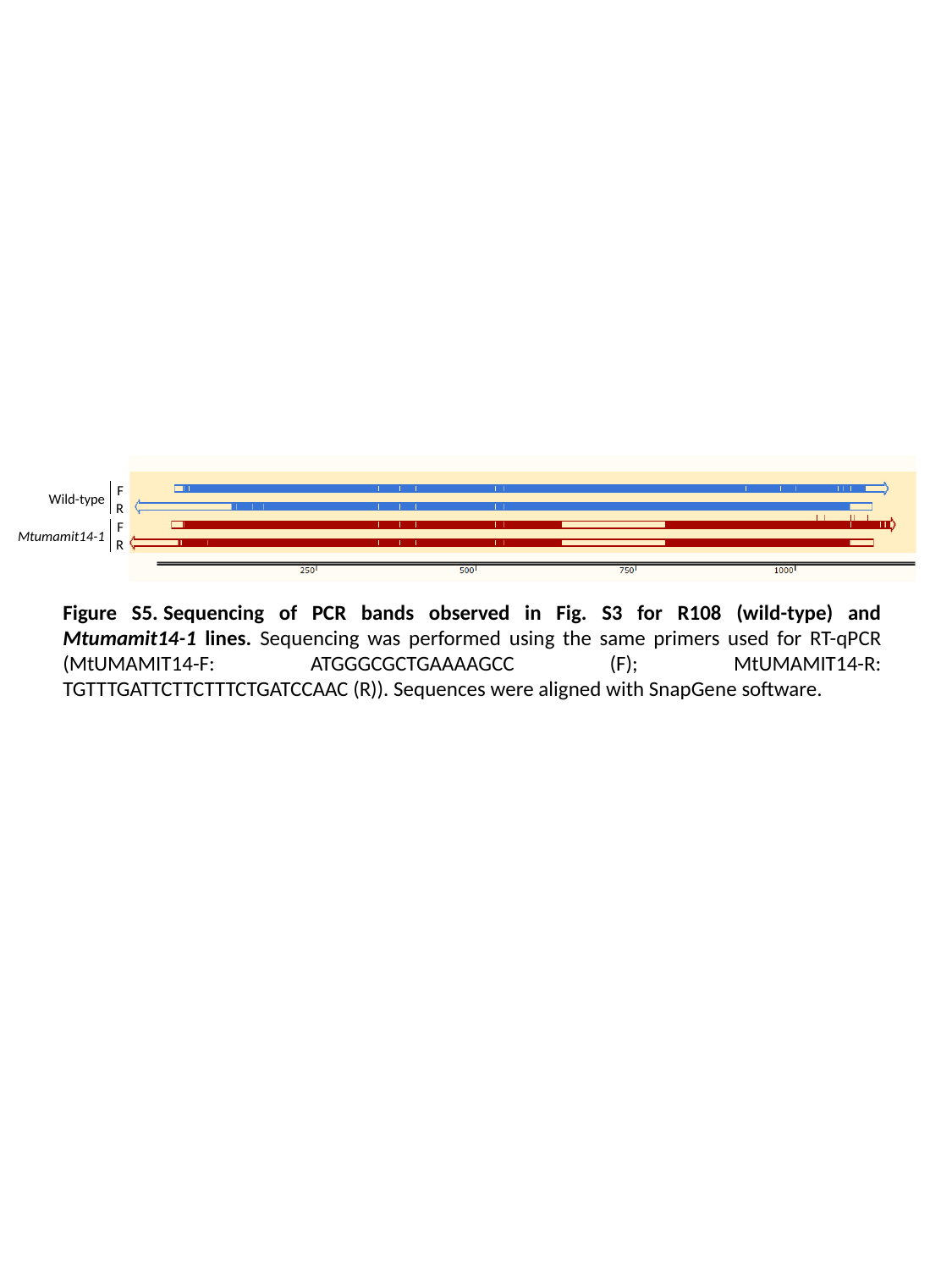

F
Wild-type
R
F
Mtumamit14-1
R
Figure S5. Sequencing of PCR bands observed in Fig. S3 for R108 (wild-type) and Mtumamit14-1 lines. Sequencing was performed using the same primers used for RT-qPCR (MtUMAMIT14-F: ATGGGCGCTGAAAAGCC (F); MtUMAMIT14-R: TGTTTGATTCTTCTTTCTGATCCAAC (R)). Sequences were aligned with SnapGene software.

## Slide 6
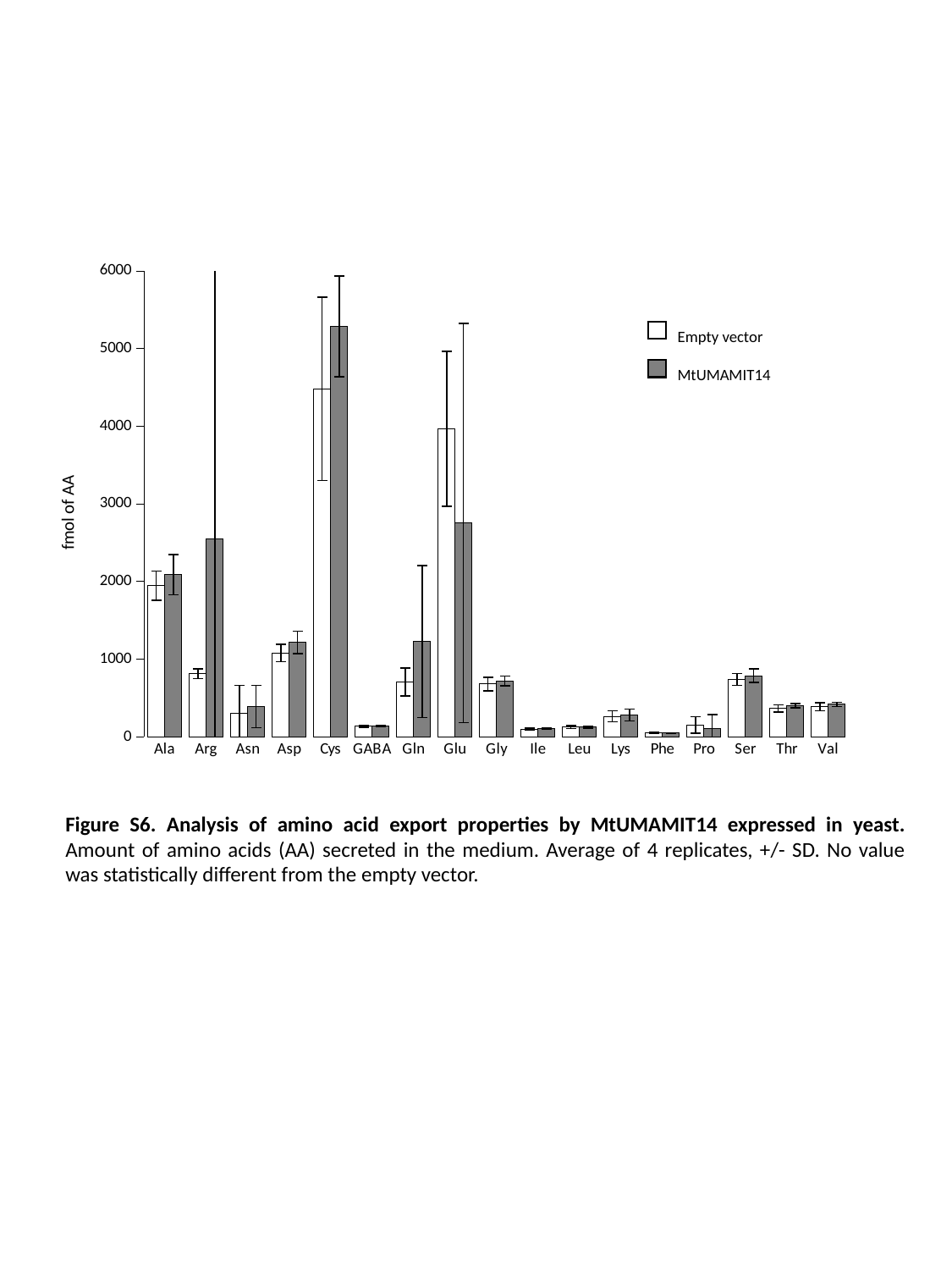

### Chart
| Category | pDR | UMAMIT9 |
|---|---|---|
| Ala | 1945.375129506654 | 2087.6147002872467 |
| Arg | 811.8025071906411 | 2551.5082364048903 |
| Asn | 307.347086714582 | 390.5499237388216 |
| Asp | 1077.9760535131911 | 1213.5525658356307 |
| Cys | 4481.0514441001615 | 5285.220174343067 |
| GABA | 135.60652959315752 | 137.5878377300722 |
| Gln | 706.4031290419537 | 1226.5315526989166 |
| Glu | 3967.287166546611 | 2752.8883034860514 |
| Gly | 678.9790217506959 | 718.5222476944825 |
| Ile | 101.01909423701444 | 103.34030283053912 |
| Leu | 125.85354381183058 | 126.1294862219672 |
| Lys | 264.00153193803476 | 280.9409703097003 |
| Phe | 51.95490894707713 | 47.33030737239554 |
| Pro | 151.44707406683546 | 104.81409722187118 |
| Ser | 740.3888391491223 | 786.5508508061382 |
| Thr | 367.1962946142006 | 400.0806887535828 |
| Val | 386.4225651713173 | 417.8332764078891 |Empty vector
MtUMAMIT14
Figure S6. Analysis of amino acid export properties by MtUMAMIT14 expressed in yeast. Amount of amino acids (AA) secreted in the medium. Average of 4 replicates, +/- SD. No value was statistically different from the empty vector.

## Slide 7
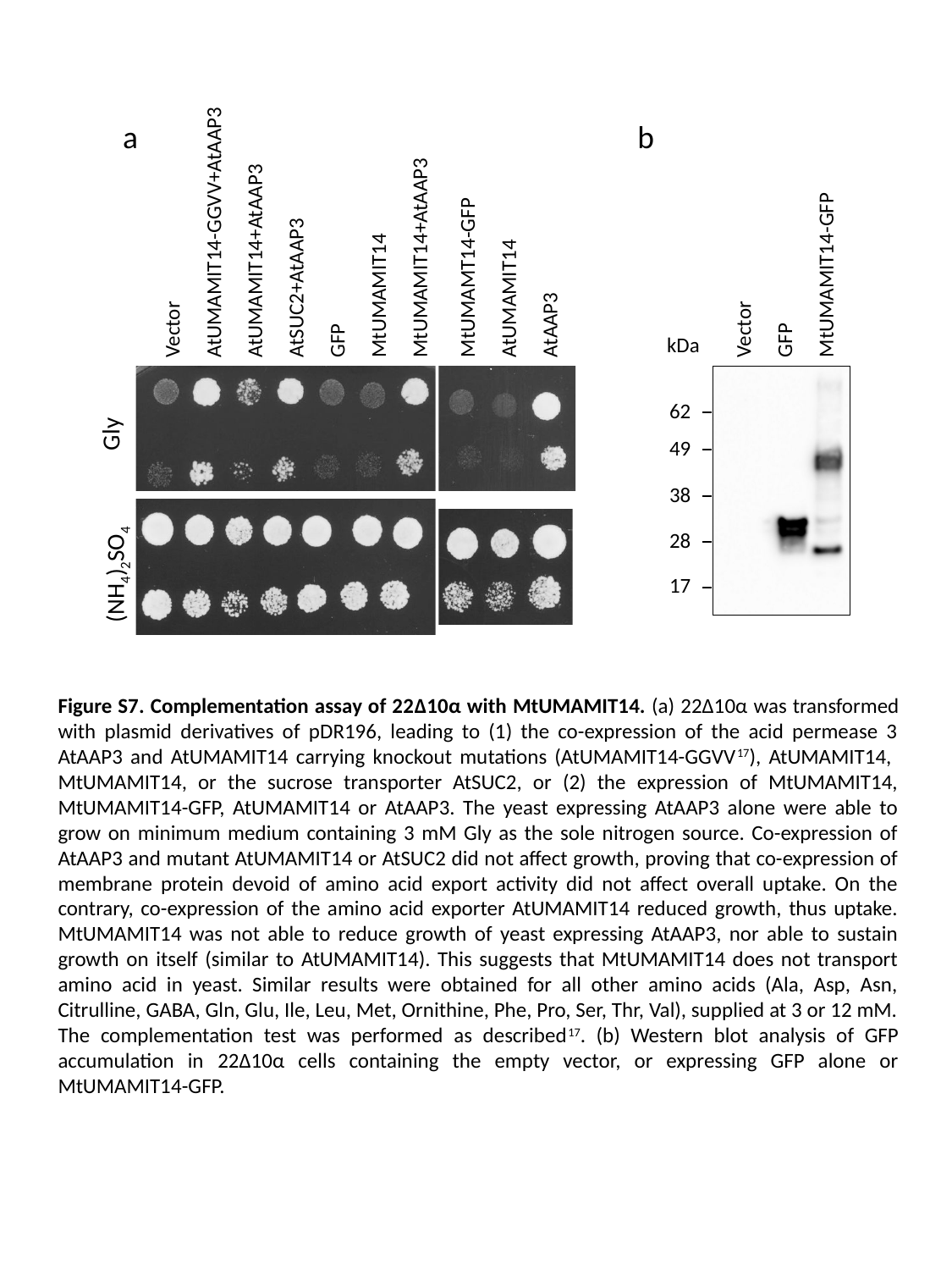

Vector
AtUMAMIT14-GGVV+AtAAP3
AtUMAMIT14+AtAAP3
AtSUC2+AtAAP3
GFP
MtUMAMIT14
MtUMAMIT14+AtAAP3
a
b
Vector
GFP
MtUMAMIT14-GFP
MtUMAMT14-GFP
AtUMAMIT14
AtAAP3
kDa
62
Gly
49
38
28
(NH4)2SO4
17
Figure S7. Complementation assay of 22Δ10α with MtUMAMIT14. (a) 22Δ10α was transformed with plasmid derivatives of pDR196, leading to (1) the co-expression of the acid permease 3 AtAAP3 and AtUMAMIT14 carrying knockout mutations (AtUMAMIT14-GGVV17), AtUMAMIT14, MtUMAMIT14, or the sucrose transporter AtSUC2, or (2) the expression of MtUMAMIT14, MtUMAMIT14-GFP, AtUMAMIT14 or AtAAP3. The yeast expressing AtAAP3 alone were able to grow on minimum medium containing 3 mM Gly as the sole nitrogen source. Co-expression of AtAAP3 and mutant AtUMAMIT14 or AtSUC2 did not affect growth, proving that co-expression of membrane protein devoid of amino acid export activity did not affect overall uptake. On the contrary, co-expression of the amino acid exporter AtUMAMIT14 reduced growth, thus uptake. MtUMAMIT14 was not able to reduce growth of yeast expressing AtAAP3, nor able to sustain growth on itself (similar to AtUMAMIT14). This suggests that MtUMAMIT14 does not transport amino acid in yeast. Similar results were obtained for all other amino acids (Ala, Asp, Asn, Citrulline, GABA, Gln, Glu, Ile, Leu, Met, Ornithine, Phe, Pro, Ser, Thr, Val), supplied at 3 or 12 mM. The complementation test was performed as described17. (b) Western blot analysis of GFP accumulation in 22Δ10α cells containing the empty vector, or expressing GFP alone or MtUMAMIT14-GFP.
